# Supplementary material for: The dual life of disordered lysine-rich domains of snoRNPs in rRNA modification and nucleolar compaction
Source: Nat Commun. 2024 Oct 31;15:9415. doi: 10.1038/s41467-024-53805-1 (PMC11528048; doi:10.1038/s41467-024-53805-1)
Supplement: Supplementary file 1 — Supplementary Information [file 41467_2024_53805_MOESM1_ESM.pdf]

A

Protists

Dictyostelium discoideum	(442)-DEKPVPKKEKKDKKEKKKDSSDDESEEEKSSKKDKKEKKEKKEKKEKKSSKDDSDDESSKKEKKDKKRKKSSDKDSDSEKESSDKKDKKDKKEKKKSN*
Symbiodinium sp.	(436)-GGADVDASDDSDDGTDVVKSAKKAKKAKKEKKEKKVKSAKKEKKAKKRSLETSPGSAAADDKTKSSKKKSRKD*
Euglena gracilis	(448)-PPKVRIEELAYAEAAKKREREAAGEDEKDAKKAKKEKKEKKEKKEKKEKAEKTA*
# *Giardia intestinalis*	(443)-SPVNEEKSSGHRKEHKKEHKKDDKKEHKKEDKKDDKKDHKKDGKKSRSKDRKEDQDAK*

Fungi (Cbf5)

Saccharomyces cerevisiae	(401)-TKETEEEPKKAKEDSLIKEVETEKEEVKEDDSKKEKKEKKDKKEKKEKKEKKDKKEKKEKKEKKRKSEDGDSEEKKSKKSKK*
Schizosaccharomyces pombe	(405)-PVVAPAAPTVEAEVNGVEDSKKRKSVESSEKDEDEAAKKEEKRRKKEAKKEKKEKKEKKEKKEKKKKSE*
Fusarium oxysporum	(418)-ASEAEDTPMEEAATPSSPAADDSKDKKRKKHDGETAEEKAERKRRKAEKKAAKAAKKAAKGEAMDSDSD*

Plants (Cbf5)

Arabidopsis thaliana	(437)-EARKRKHDDSSDSPAPVTTKKSKTKEVEGEEAEEKVKSSKKKKKKDKEEEKEEEAGSEKKEKKKKKDKKEEVIEEVASPKSEKKKKKKSKDTEAAVDAE DESAAEKSEKKKKKKDKKKKNKDSEDDEE*

Animals (Dkc1)

Homo sapiens	(442)-KTAKRKRESESESDETPPAAPQLIKKEKKKSKKDKKAKAGLESGAEPGDGDSDTTKKKKKKKKAKEVELVSE*
Gallus gallus	(416)-YRESSKKEAAKVPQAVSEVERAPKRKRESESENEAVSPPPSPATPPPEELSKKEKKKKKKEKKAKEAAESGEEQVEVISESSAKKKKKKKKQKEVEESSE*
Rattus norvegicus	(442)-SSKKATAAEATPGPGVTADAASIVKRKRDSDSDADEATPTTTPRVKKEKKKKKEKADGGEEAAEDGDGDATRKKKKKKARAAEELSG*
Danio rerio	(411)-YVDYSTTKVKKGGEASAKRKRDESGSEGEAAAAAQDTSKTEEESKKEKKKKKKEKKQKLAEEAAAEAPAEEETEVTESAKKKKKKKKAKETEADSD*
Caenorhabditis elegans	(385)-KFGKPNDTTPKSWAKEYVQTSTKKEVKKEETPDEEEEEAPKKKSKKSKKQESSDSDSD*
Drosophila melanogaster	(426)-VSPTNGSSEPSKRKLSTSSVEETAAAAVSEETPSKDKKKKKKKHKGDEEAPEAAEEEAEPVEKEKKKKKKKDKDRDRDEAQE*

B

	Kappa	FCR	NCPR	Disorder Promoting
Nop56 *(Sc)*	0.133	0.873	0.175	0.968
Nop58 *(Sc)*	0.152	0.892	0.135	1
Cbf5 *(Sc)*	0.1	0.744	0.078	0.956
Nop56 *(Hs)*	0.371	0.456	0.089	0.848
Nop58 *(Hs)*	0.344	0.544	0.074	0.831
Cbf5/Dyskerin *(Hs)*	0.294	0.438	0.085	0.838
Nop56 *(Sp)*	0.355	0.615	0.062	0.862
Nop58 *(Sp)*	0.338	0.662	0.122	0.919
Cbf5 *(Sp)*	0.133	0.554	0.089	0.839

C

	Length	Kappa	FCR	NCPR	Hydropathy	Disorder promoting
1_Caenorhabditis elegans	59	0.299	0.473	0.065	2.859	0.806
2_Fusarium oxysporum	69	0.277	0.522	0	2.78	0.971
3_Dictyostelium discoideum	99	0.133	0.788	0.121	1.392	0.98
4_Symbiodinium sp.	73	0.309	0.548	0.164	2.77	0.932
5_Gallus gallus	100	0.307	0.51	0.07	2.712	0.89
6_Rattus norvegicus	87	0.389	0.471	0.057	3.056	0.943
7_Arabidopsis thaliana	128	0.285	0.68	0.055	2.138	0.938
8_Drosophila melanogaster	76	0.342	0.592	0.013	2.422	0.947
9_Euglena gracilis	54	0.09	0.667	0.111	2.439	0.926

D

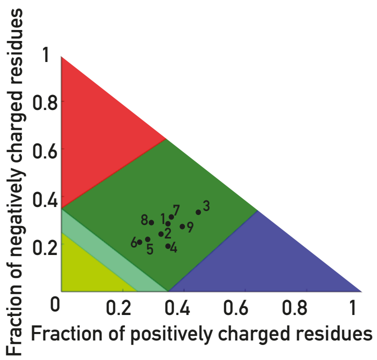

E

Nucleus	Dbp2	RSYGGGHPRYGGGRGGRGGYGRRGGYGGGRGGYGGNRQRDGGWGNRGRSNY
	Npl3	RGRGGFRGGFRGGFRGGFSRGGFGGPRGGFGGPRGGYGGYSRGGYGGYSRGGY
Nucleolus	Nop1	RPGSRGGSRGGSRGGFGGRGGSRGGARGGSRGGFGGRGGSRGGARGGSRGGF
	Gar1	RGGASMGRGGSRGGFRGGRGGSSFRGGRGGSSFRGGSRGGSFRGGSRGGSRGGF

F

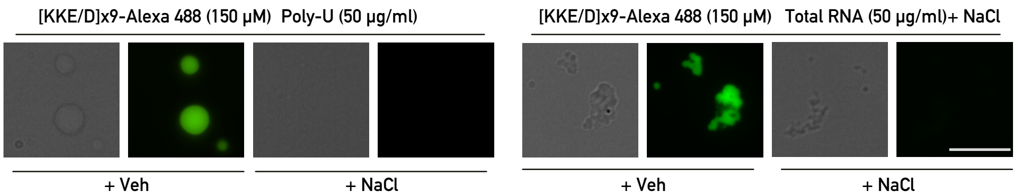

Supplementary Figure 1: A lysine-rich IDR is present in a subset of abundant, early-acting ribosome biogenesis factors.

(A) Amino acid sequence of the KKE/D domains of Cbf5 in different eukaryotes referenced in UniProt. The numbers correspond to the amino acid positions in the full-length sequence. *: C-terminus of the proteins. The name of Cbf5 orthologs associated with different groups of eukaryotes (Fungi, Plants, Animals) is indicated. There is no common name of Cbf5 orthologs in Protists (*Dictyostelium discoideum*: Nola4, *Symbiodinium sp.*: UniProt ID A0A812MXA0, *Euglena gracilis*: Cbf5). #: the KKE/D domain of Nop56 instead of Cbf5 is presented for *Giardia intestinalis* because the annotated Cbf5 ortholog in this organism lacks a (clear) KKE/D domain (UniProt ID: V6U394). **(B)** The Kappa, Frequency of Charged Residues (FCR), Net Charge Per Region (NCPR), Ratio of Disorder Promoting Residues (Disorder Promoting) values are indicated for the lysine-rich IDRs of Nop56, Nop58, and Cbf5/Dyskerin in *S. cerevisiae* (*Sc*), *H. sapiens* (*Hs*) and *S. pombe* (*Sp*). **(C)** The Kappa, Frequency of Charged Residues (FCR), Net Charge Per Region (NCPR), Hydropathy, Ratio of Disorder Promoting Residues (Disorder Promoting) values are indicated for the lysine-rich IDRs of Cbf5 in different eukaryotic species. **(D)** Diagram of states for the KKE/D domains of Cbf5 and its eukaryotic homologs detailed in (C). The red and blue areas correspond to strong polyelectrolyte features with FCR > 0.35 and NCPR > 0.3, exhibiting coil-like conformations. The dark green area corresponds to strong polyampholytes that form non-globular conformations (coil-like, hairpin-like, or hybrids), whereas the light green area corresponds to IDRs predicted to form globular or tadpole-like conformations. **(E)** Amino acid sequence and composition of the GAR domains of the abundant nuclear (Dbp2, Npl3) and nucleolar (Nop1, Gar1) proteins. **(F)** Coacervates were formed *in vitro* by incubating for 15 min at room temperature a mix of [KKE/D]x9-Alexa 488 peptide and either poly-uridine (poly-U) RNAs or total RNA from *S. cerevisiae*. Coacervate suspension was next observed 60 min following addition of a Tris-buffered solution containing NaCl (+ NaCl, 300 mM final NaCl concentration) or the Tris-buffered solution without NaCl (+ Veh). Scale bar = 10 µm.

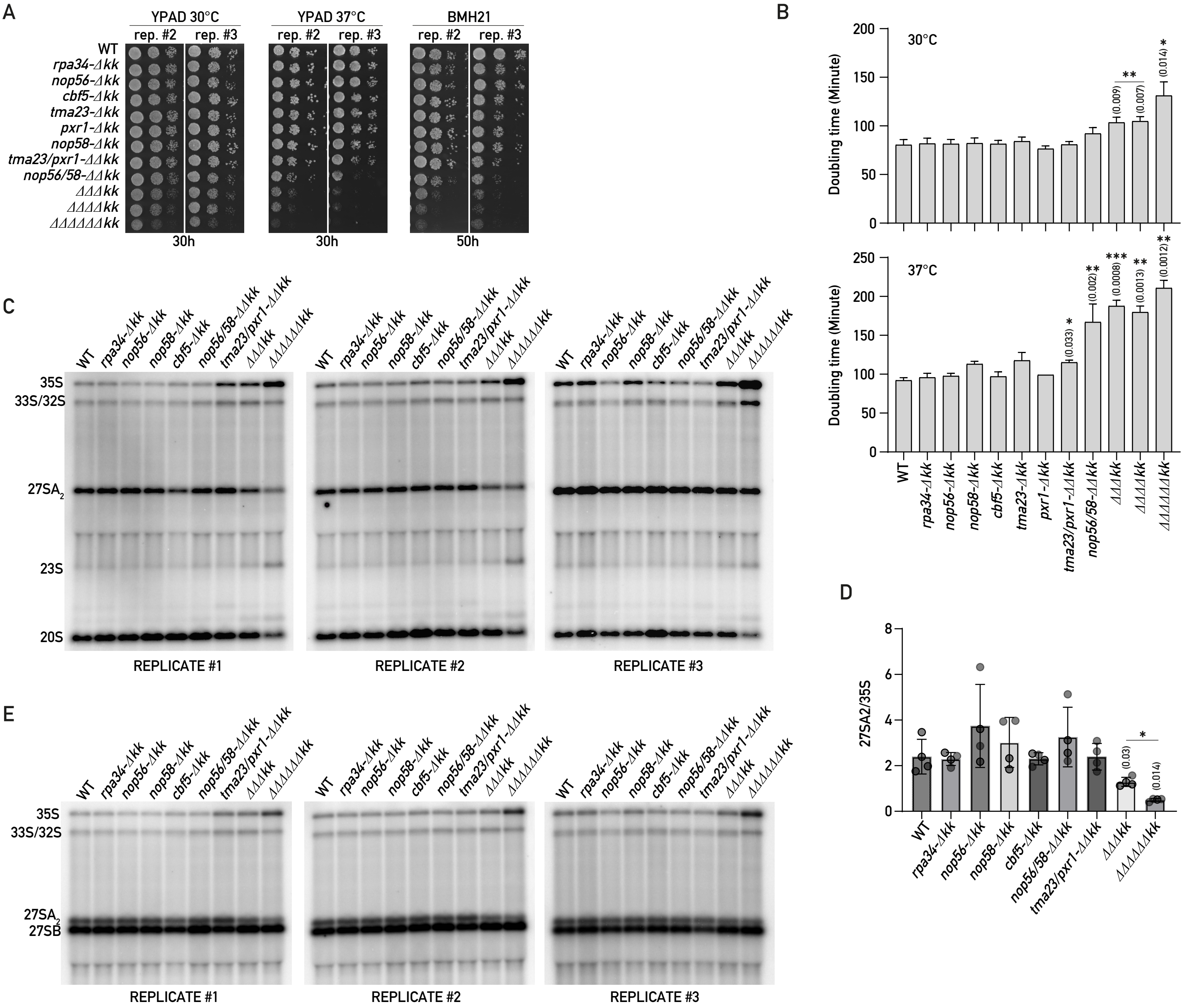

Supplementary Figure 2: snoRNP KKE/D domains are collectively required for optimal growth, pre-rRNA processing and rRNA modification.

(A) Two replicates (rep#1, #2) of tenfold serial dilutions of wild-type or mutant strains bearing individual or multiple deletions of KKE/D domains (*-Δkk*) were grown in YPAD medium for 30 h at 30°C or 37°C or in the presence of a sub-lethal dose (15 µM) of BMH-21 for 50 h. *rpa34-Δkk* (Rpa34-(1-186)), *nop56-Δkk* (Nop56-(1-441)), *cbf5-Δkk* (Cbf5-(1-402)), *tma23-Δkk* (Tma23-(1-141)), *pxr1-Δkk* (Pxr1-(1-149)), *nop58-Δkk* (Nop58-(1-438)), *ΔΔΔkk (nop56-Δkk, nop58-Δkk, cbf5-Δkk), ΔΔΔΔkk (nop56-Δkk, nop58-Δkk, cbf5-Δkk, rpa34-Δkk), ΔΔΔΔΔΔkk (nop56-Δkk, nop58-Δkk, cbf5-Δkk, rpa34-Δkk, pxr1-Δkk, tma23-Δkk)*. **(B)** The doubling time of each strain has been measured in triplicate experiments by following the optic density (OD_{600}) of liquid cultures in YPD at 30°C (top panel) or 37°C (bottom panel). A median of the three technical and biological replicates is shown. Unpaired two-tailed t-test analysis was used for statistics. Data are presented as mean values +/− SD. Significant differences are indicated by stars and with the p-value on the graph. n = 3 biologically independent experiment. **(C)** Three technical and biological replicates of steady-state levels of rRNA precursors in the wild-type (WT) strain and the indicated KKE/D domain mutant strains. Total RNAs extracted from these strains were analyzed by Northern blotting using radiolabeled probes (23S.1 + 20S.3, Supplemental Data 8) detecting the indicated precursors. **(D)** Quantification of the northern blot signals obtained for the hybridizations shown in (C) using PhosphorImager data and the MultiGauge software. The histogram represents the $27SA_2/35S$ ratio obtained in four technical and biological replicates in the wild-type (WT) strain and the indicated KKE/D domain mutant strains. Statistically significant differences were determined using unpaired two-tailed Student's t-test. Significant differences are indicated by stars and with the p-value on the graph. n = 4 biologically independent experiment. **(E)** Three technical and biological replicates of steady-state levels of rRNA precursors in the wild-type (WT) strain and the indicated KKE/D domain mutant strains. Total RNAs extracted from these strains were analyzed by Northern blotting using the radiolabeled rRNA2.1 probe (Supplemental Data 8) detecting the indicated precursors.

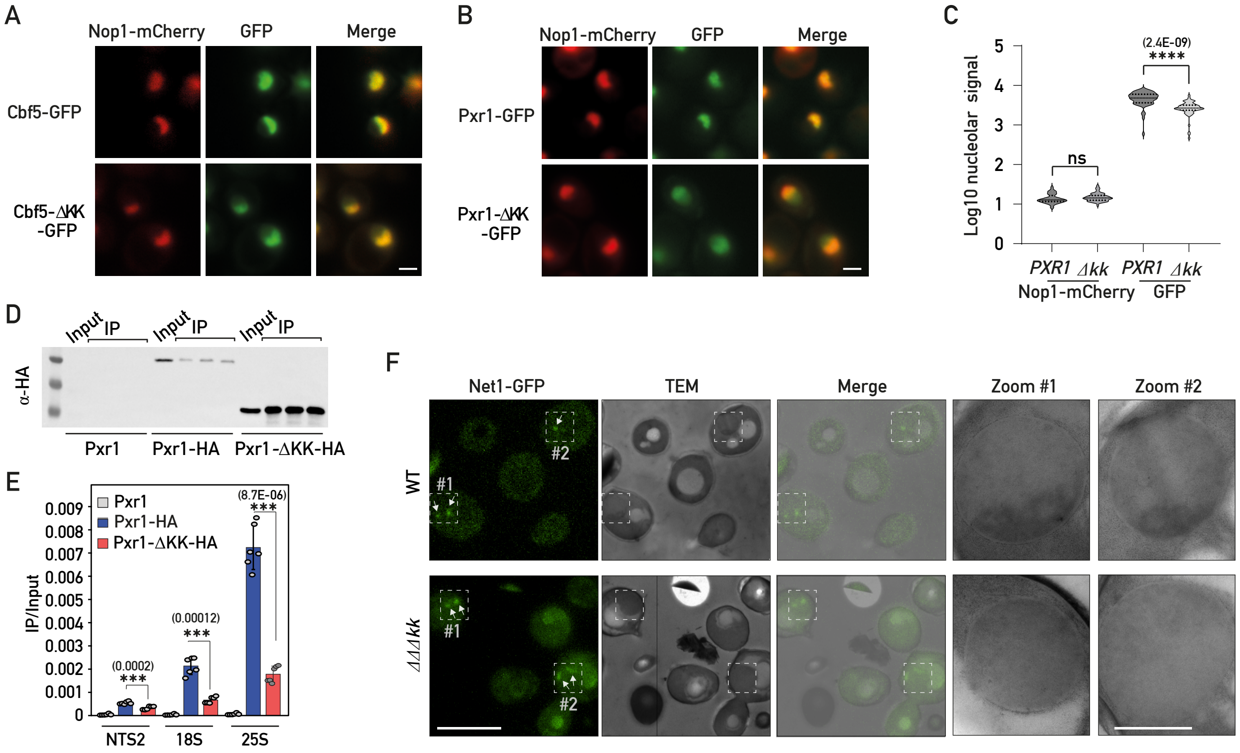

Supplementary Figure 3: The KKE/D domain is essential for recruitment to the vicinity of rDNA genes.

(A) Fluorescence microscopy analyses of cells expressing Nop1-mCherry and either Cbf5-GFP or Cbf5-ΔKK-GFP. Merge: overlay of both fluorescent signals. Scale bar = 2 µm. **(B)** Same legend as in (A) for Pxr1-GFP or Pxr1-ΔKK-GFP. Scale bar = 2 µm. **(C)** Quantification of the nucleolar Nop1-mCherry and GFP signals (Log10) in *PXR1*-GFP (*PXR1*; *n* = 51) and *pxr1-Δkk*-GFP (Δ*kk*; *n* = 51) strains. *p-values* were calculated with unpaired two-tailed Welch's t-test. *n* = number of cells pooled from 3 biologically independent replicates. **(D)** Western blot analysis using anti-HA antibodies showing the IP efficiencies of Pxr1 (no tag control), Pxr1-HA or Pxr1-ΔKK-HA in the ChIP-qPCR experiments shown in (E). Three technical IP replicates are shown for each condition as well as the corresponding input sample. **(E)** Pxr1 occupancy on rDNA genes at 18S, 25S or intergenic (NTS2) regions in strains expressing Pxr1 (no tag control providing the background binding), Pxr1-HA and Pxr1-ΔKK-HA evaluated by ChIP-qPCR. Immunoprecipitations were performed using anti-HA antibodies. DNA occupancy was defined as the ratio between the immunoprecipitation (IP) and the input signals measured by qPCR. Unpaired two-tailed t-test analysis was used for statistics. Data are presented as mean values +/− SD. Significant differences are indicated by stars and with the *p-value* on the graph. n = 6 biologically independent experiment. **(F)** Representative images of wild-type (WT) or *cbf5-Δkk*, *nop56-Δkk, nop58-Δkk (ΔΔΔkk)* cells expressing Net1-GFP grown exponentially and analyzed for ultrastructural studies by Correlative Light and Electron Microscopy (CLEM) method using cryofixed, cryosubstituted cells. Representative sections of wild-type and mutant cell nuclei observed by fluorescence microscopy (Net1-GFP) are presented (left panel). Two specific WT or *ΔΔΔkk* nucleus sections showing Net1-GFP signal were next analyzed for ultrastructural studies by transmission electron microscopy (TEM, scale bar = 15 µm for Net1-GFP, TEM and Merge images). Merge: overlay of the fluorescent signals and TEM images. Zooms of the cells within the indicated dotted squares of TEM images are shown on the right (scale bar = 2 µm).

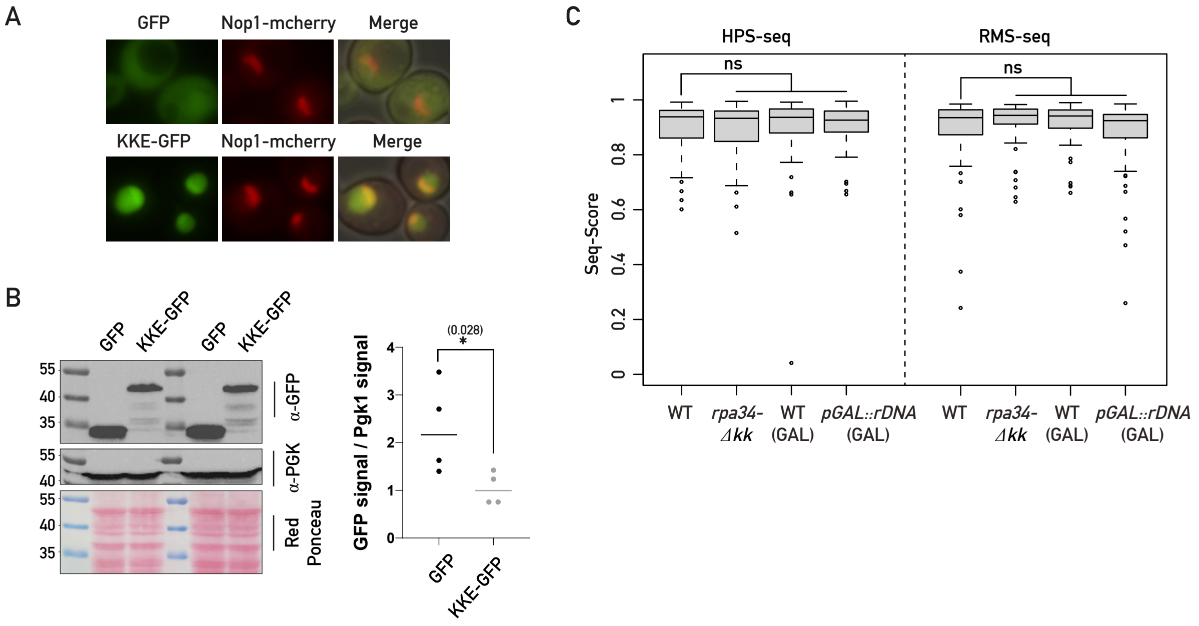

Supplementary Figure 4: The KKE/D domain is sufficient to promote efficient targeting close to transcribed rDNA genes.

(A) Strains expressing Nop1-mCherry and the KKE/D-GFP construct (KKE-GFP) or GFP alone were grown exponentially and cells were analyzed by fluorescence microscopy. **(B)** Western blotting experiments using anti-GFP antibodies and extracts from cells expressing the KKE/D-GFP construct (KKE-GFP) or GFP alone as in (A). The anti-Pgk1 signals and the staining of total proteins with Ponceau red served as loading controls. Right panel: quantification of GFP signals normalized by Pgk1 signals. Paired two-tailed t-test analysis was used for statistics. Significant differences are indicated by stars and with the *p-value* on the graph. n = 4 biologically independent experiment. **(C)** Density plots showing HPS scores (left panel) and RMS scores (right panel) for all rRNA modification sites (n =47 for HydraPsi-Scores and n =55 for RiboMeth-Score) in wild-type (WT) and *rpa34-Δkk* cells grown on glucose or in wild-type (WT (GAL)) and *rdn1Δ pGAL::rDNA rpa135Δ* (*pGAL::rDNA*) cells grown on galactose. These latter cells lack rDNA chromosomal repeats and the essential Rpa135 RNAPI subunit; they express rRNAs from a multicopy plasmid under the control of a galactose-inducible promoter (RNAPII). HydraPsi-Score (HPS-Score) and RiboMeth-Score indicate the fraction of uridine isomerisation and 2′-*O*-methylation at each rRNA modification site, respectively. *p-values* were calculated with unpaired two-samples Wilcoxon test (ns = no significance).

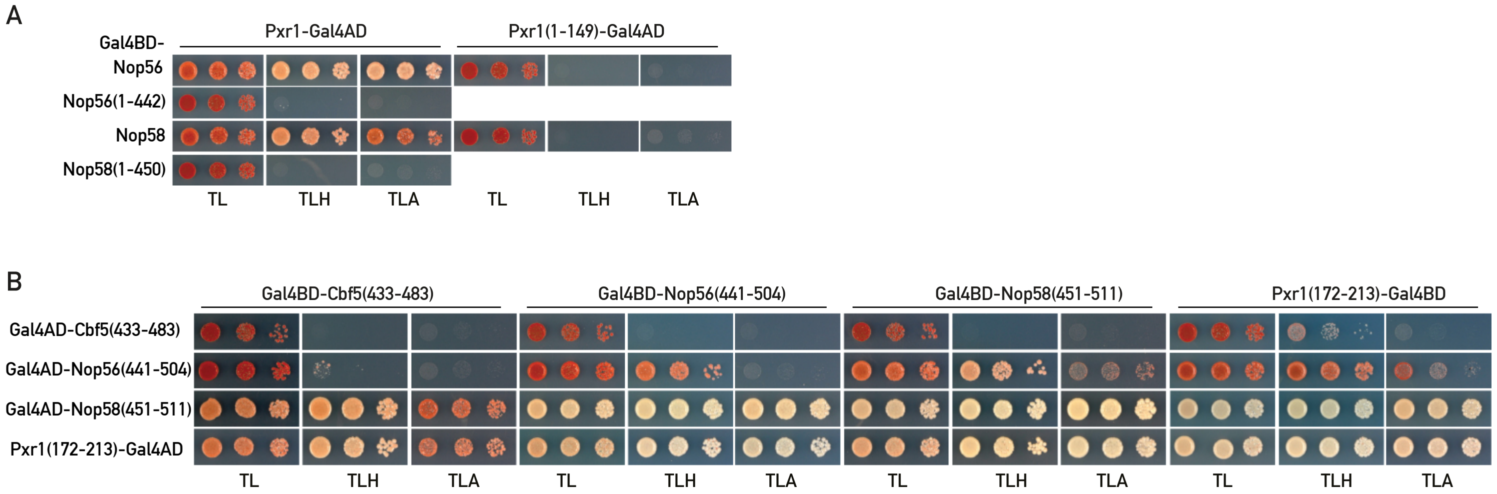

Supplementary Figure 5: KKE/D domains interact in a homo- and heterotypic manner.
(A) Y2H assays using the indicated combinations of the Gal4 DNA-binding domain (Gal4BD) fused to full-length Nop56, Nop56 lacking its KKE/D domain (Nop56(1-442)), full-length Nop58 or Nop58 lacking its KKE/D domain (Nop58(1-450)) and the Gal4 activation domain (Gal4AD) fused to full-length Pxr1 or Pxr1 lacking its KKE/D domain (Pxr1(1-149)). *n* = 3 biologically independent experiment. **(B)** Y2H assays using the indicated combinations of the Gal4 DNA-binding domain (Gal4BD) fused to the KKE/D repeats of Cbf5 (Cbf5(433-483)), the KKE/D domain of Nop56 (Nop56(441-504)), the KKE/D domain of Nop58 (Nop58(451-511)), or the KKE/D repeats of Pxr1 (Pxr1(172-213)) and the Gal4 activation domain (Gal4AD) fused to the KKE/D repeats of Cbf5 (Cbf5(433-483)), the KKE/D domain of Nop56 (Nop56(441-504)), the KKE/D domain of Nop58 (Nop58(451-511)), or the KKE/D repeats of Pxr1 (Pxr1(172-213)). *n* = 3 biologically independent experiment.

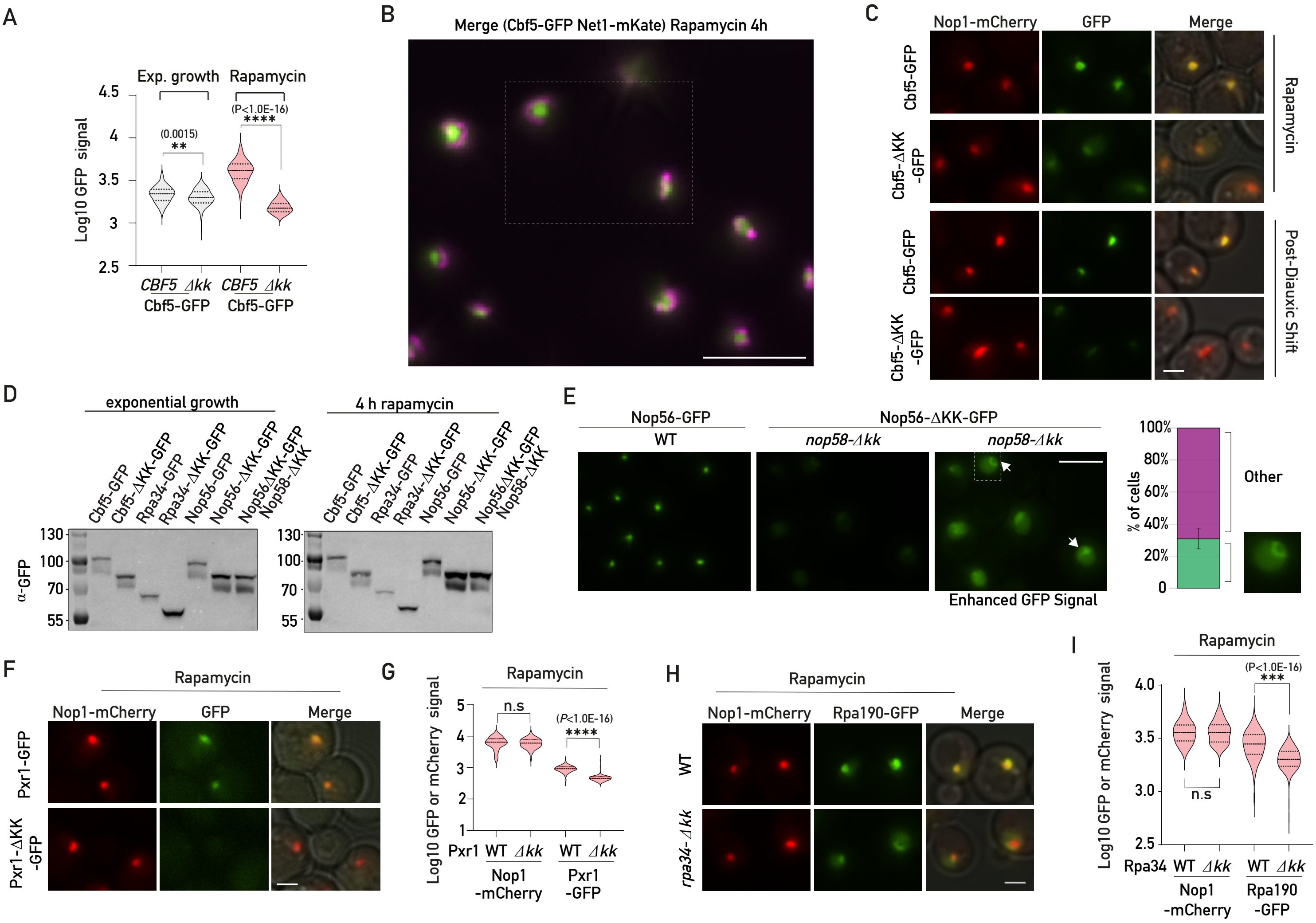

Supplementary Figure 6: KKE/D domains are essential for nucleolar compaction and sequestration of associated factors in a specific subnucleolar area following TORC1 inactivation.

(A) Quantification of maximum nuclear GFP and mKate signals (Log10) in rapamycin-treated (*n* =120) or untreated cells (*n* =120) described in Figure 6A. *CBF5*-GFP (*CBF5*) and *cbf5-Δkk*-GFP (Δ*kk*) cells expressing Net1-mKate were grown exponentially (Exp. growth) or treated for 4 h with rapamycin (Rapamycin). *p-values* were calculated with unpaired two-tailed Welch's t-test. *n* = number of cells pooled from 3 biologically independent replicates. **(B)** Fluorescence microscopy analyses of cells expressing Cbf5-GFP (Green) and Net1-mKate (Magenta) following rapamycin treatment. The dotted rectangle corresponds to the cells shown in Figure 6A. Scale bar = 5 µm. **(C)** Fluorescence microscopy analyses of cells expressing Nop1-mCherry and either Cbf5-GFP or Cbf5-ΔKK-GFP treated with rapamycin (Rapamycin) or in the post-diauxic growth phase. Scale bar = 2 µm. **(D)** Western blotting experiment using anti-GFP antibodies and protein extracts from cells expressing GFP-tagged versions of wild-type Cbf5 (Cbf5-GFP), Rpa34 (Rpa34-GFP) or Nop56 (Nop56-GFP), mutants lacking the KKE/D domain (Cbf5-ΔKK-GFP, Rpa34-ΔKK-GFP, Nop56-ΔKK-GFP) or mutants lacking the KKE/D domains of Nop56 and Nop58 ((Nop56-ΔKK-GFP Nop58-ΔKK). Cells were grown exponentially (Exponential growth) or treated for 4 h with rapamycin (Rapamycin). **(E)** Fluorescence microscopy analyses of rapamycin-treated wild-type or *nop58-Δkk* cells expressing Nop56-GFP or Nop56-ΔKK-GFP as in Figure 6D. Scale bar = 5 µm.16-bit brightness levels have been increased to specifically visualize the GFP signal in *nop56-Δkk*-GFP *nop58-Δkk* cells (Enhanced GFP Signal). The percentage of cells (*n* = 120) showing a circular GFP-depleted area pattern (arrow) in comparison to all other GFP patterns (Other) was manually counted in a series of four independent experiments. Such a GFP pattern is absent in all wild-type cells examined (*n* = 120). Data are presented as mean values +/− SD. **(F)** Fluorescence microscopy analyses of cells expressing Nop1-mCherry and either Pxr1-GFP or Pxr1-ΔKK-GFP treated with rapamycin. Scale bar = 2 µm. **(G)** Quantification of nucleolar Nop1-mCherry and GFP signals (Log10) in cells inspected in (F). (WT; *n* = 185, *Δkk*; *n* = 217). *p-values* were calculated with unpaired two-tailed Welch's t-test. *n* = number of cells pooled from 3 biologically independent replicates. **(H)** Fluorescence microscopy analyses of rapamycin-treated wild-type (WT) or *rpa34-Δkk* cells expressing Nop1-mCherry and Rpa190-GFP. Scale bar = 2 µm. **(I)** Quantification of nucleolar Nop1-mCherry and Rpa190-GFP signals (Log10) in cells inspected in (H). (WT; *n* = 176, *Δkk*; *n* = 138). *p-values* were calculated with unpaired two-tailed Welch's t-test. *n* = number of cells pooled from 3 biologically independent replicates.

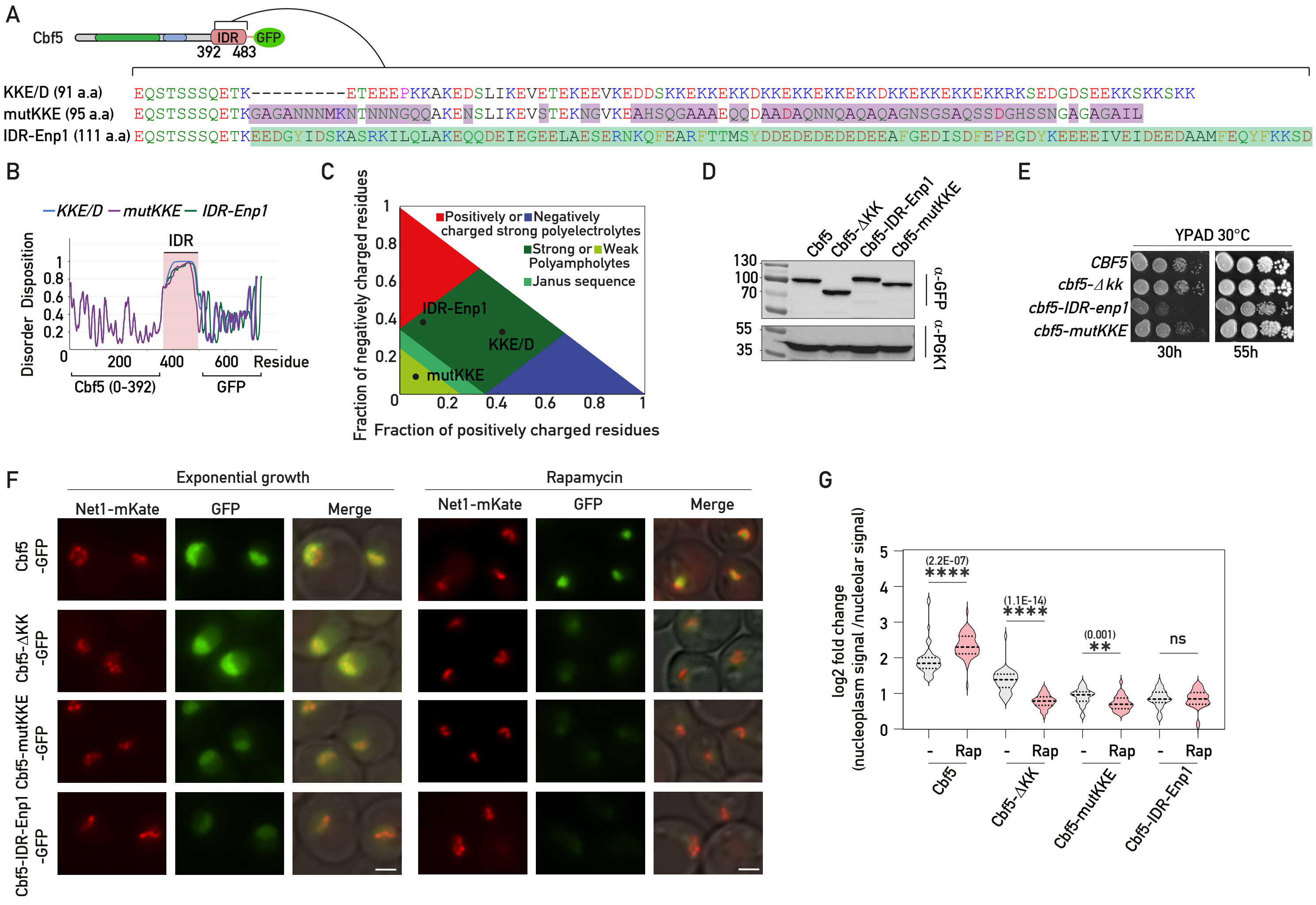

Supplementary Figure 7: KKE/D domain has specific properties essential for nucleolar organization.

(A) Schematic representation of Cbf5 KKE/D domain mutations. The natural IDR sequence of Cbf5 and mutated sequences (IDR-enp1 or mutKKE) are shown. Mutated IDRs were inserted in Cbf5 after amino acid 403 in place of the KKE/D domain. The magenta shaded areas in mutKKE indicate mutated residues. Most of the charged amino acids of the KKE/D domain have been replaced by G, A, Q, N or S residues to preserve the flexibility of this IDR and to avoid non-physiological large repetitions of similar amino acids. The green shaded area in the IDR-enp1 sequence corresponds to the amino acid sequence 48-148 of the Enp1 protein. **(B)** Prediction of disorder tendency of KKE/D mutants with PONDR (Peng et al., 2006). **(C)** Diagram of states for IDRs presented in A. **(D)** Western blotting experiment using anti-GFP or anti-Pgk1 antibodies and protein extracts from cells expressing wild-type Cbf5 (Cbf5), the truncated version lacking the KKE/D domain (Cbf5-ΔKK*)* or Cbf5 with mutated versions of the KKE/D domain (Cbf5-IDR-Enp1, Cbf5-mutKKE) fused to GFP. **(E)** Tenfold serial dilutions of wild-type cells expressing wild-type Cbf5 (*CBF5*), the truncated version lacking the KKE/D domain (*cbf5-Δkk)* or Cbf5 with mutated versions of the KKE/D domain (*cbf5-IDR-enp1*, *cbf5-mutKKE*) fused to GFP grown in YPAD medium for 30 h or 55 h. *n* = 3 biologically independent experiment. **(F)** Fluorescence microscopy analyses of untreated (Exponential growth) or rapamycin-treated (Rapamycin) cells expressing wild-type Cbf5 (Cbf5-GFP), the truncated version lacking the KKE/D domain (Cbf5-ΔKK-GFP*)* or Cbf5 with mutated versions of the KKE/D domain (Cbf5-IDR-Enp1-GFP, Cbf5-mutKKE-GFP) fused to GFP. Merge: overlay of fluorescent and transmission signals. Scale bars = 2 µm. **(G)** Quantification of log2 ratios between nucleolar and nucleoplasmic GFP signals in exponential growth (-) conditions (Cbf5 (*n* = 52), Cbf5-ΔKK (*n* = 41), Cbf5-mutKKE (*n* = 29), Cbf5-IDR-Enp1 (*n* = 40)) or following rapamycin (Rap) treatment (Cbf5 (*n* = 56), Cbf5-ΔKK (*n* = 45), Cbf5-mutKKE (*n* = 28), Cbf5-IDR-Enp1 (*n* = 36)). *p-values* were calculated with unpaired two-tailed Welch's t-test. *n* = number of cells pooled from 3 biologically independent replicates.

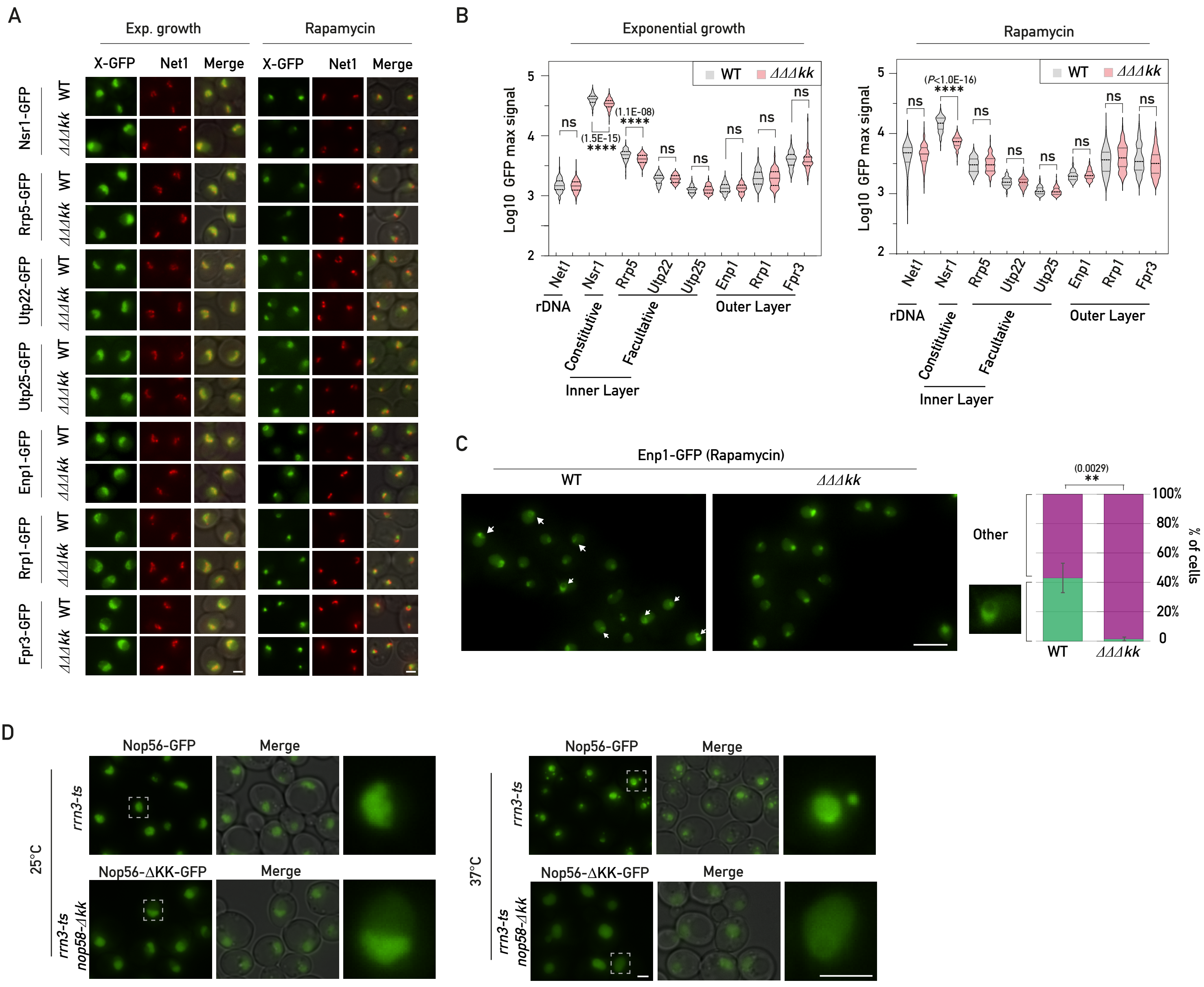

Supplementary Figure 8: KKE/D domains are essential for nucleolar compaction and sequestration of associated factors in a specific subnucleolar area following TORC1 inactivation.

(A) Fluorescence microscopy analyses of cells expressing Net1-mKate (rDNA staining) and the indicated proteins fused to GFP (X-GFP) grown exponentially (Exp. growth, left panel) or treated for 4 h with rapamycin to inactivate TORC1 (Rapamycin, right panel). Merge: overlay of fluorescent and transmission signals. Scale bars = 2 µm. **(B)** Quantification of maximum nuclear GFP and mKate signals in rapamycin (Rap) treated cells (Net1 (*n* =98), Nsr1 (*n* =136), Rrp5 (*n* =180), Utp22 (*n* =120), Utp25 (*n* =124), Enp1 (*n* =228), Rrp1 (*n* =101), Fpr3 (*n* =138) or untreated (-) cells (Net1 (*n* =248), Nsr1 (*n* =155), Rrp5 (*n* =114), Utp22 (*n* =99), Utp25 (*n* =144), Enp1 (*n* =248), Rrp1 (*n* =243), Fpr3 (*n* =116)) described in panel (A). *p-values* were calculated with unpaired two-tailed Welch's t-test. *n* = number of cells pooled from 3 biologically independent replicates. **(C)** Fluorescence microscopy analyses of rapamycin-treated wild-type or *ΔΔΔkk* cells expressing Enp1-GFP. Scale bar = 5 µm. The percentage of cells showing a circular GFP-depleted area pattern (arrows) in comparison to all other GFP patterns in both wild-type (*n* = 120) or *ΔΔΔkk (n* = 120) cells was manually counted in a series of four independent experiments. *p-values* were calculated with unpaired two-tailed Welch's t-test. **(D)** Fluorescence microscopy analyses of *rrn3-ts* or *rrn3-ts nop58-Δkk* cells expressing Nop56-GFP or Nop56-ΔKK-GFP, respectively, grown exponentially at 25°C (active RNAPI) or shifted for 1 h to 37°C (inactive RNAPI, no rRNA production). Zooms of the GFP signals in the indicated dotted squares are shown on the right. Scale bar = 2 µm.
