# Supplementary material for: The dual life of disordered lysine-rich domains of snoRNPs in rRNA modification and nucleolar compaction
Source: Nat Commun. 2024 Oct 31;15:9415. doi: 10.1038/s41467-024-53805-1 (PMC11528048; doi:10.1038/s41467-024-53805-1)
Supplement: Supplementary file 2 — Description of Additional Supplementary Files [file 41467_2024_53805_MOESM2_ESM.pdf]

Description of Additional Supplementary Files

File Name: Supplementary Data 1

Description: IDRs of S. cerevisiae AMFs longer than 30 amino acids (n=108) with their Frequency of Charged Residues (FCR), Net Charge per IDR as defined in (Holehouse et al., 2017), size in amino acids (a.a) and coordinates in the protein sequence defined in Zarin et al, 2021. For a given protein with several different IDRs, we only consider the largest IDR >40 amino acid.

File Name: Supplementary Data 2

Description: Dataset showing HPS scores for each pseudouridylation site in wild-type or mutant cells grown on glucose (WT, rpa34-Δkk, nop56/58-ΔΔk, cbf5-Δkk and pxr1-Δkk tma23-Δkk) or galactose (WT Gal and rdn1Δ pGAL::rDNA rpa135Δ). HPS scores for each replicate (_rep1, _rep2, _rep3) are indicated.

File Name: Supplementary Data 3

Description: Dataset showing HPS scores for each pseudouridylation site in wild-type or mutant cells grown on glucose (WT, rpa34-Δkk, nop56/58-ΔΔk, cbf5-Δkk and pxr1-Δkk tma23-Δkk) or galactose (WT Gal and rdn1Δ pGAL::rDNA rpa135Δ). HPS scores for each replicate (_rep1, _rep2, _rep3) are indicated.

File Name: Supplementary Data 4

Description: Enrichment of proteins identified by mass spectrometry in cells expressing the KKE/D domain of Cbf5 fused to BirA (Log2(BirA-KKE)) or the SV40 NLS-BirA-GFP control protein (Log2(NLS-BirA-GFP)). The difference between Log2(BirA-KKE) and Log2(NLS-BirA-GFP) (∆log2) was used to evaluate the enrichment of proteins purified upon BirA-KKE expression in comparison to background.

File Name: Supplementary Data 5

Description: List of the most efficiently biotinylated proteins (∆log2 > 1.25) and Gene Ontology associated to these genes determined using YeastMine (https://yeastmine.yeastgenome.org/yeastmine/begin.do)..

File Name: Supplementary Data 6

Description: Yeast strains used in this study.

File Name: Supplementary Data 7

Description: Plasmids used in this study.

File Name: Supplementary Data 8

Description: Primers used in this study.

File Name: Supplementary Data 8

Description: Mass spectrometry data TurboID Raw data output.

File Name: Supplementary Data 9

Description: List of each protein detected and its associated peptide count, sequence coverage, intensity and iBaq value in the purification of biotinylated proteins from cells expressing the BirA-KKE or cells expressing the NLS-BirA-GFP bait.
