# Supplementary material for: The dual life of disordered lysine-rich domains of snoRNPs in rRNA modification and nucleolar compaction
Source: Nat Commun. 2024 Oct 31;15:9415. doi: 10.1038/s41467-024-53805-1 (PMC11528048; doi:10.1038/s41467-024-53805-1)
Supplement: Supplementary file 12 — Reporting Summary [file 41467_2024_53805_MOESM12_ESM.pdf]

nature portfolio

Corresponding author(s): Dieter Kressler
Olivier Gadal
Anthony K. Henras
Benjamin Albert

Last updated by author(s): Oct 21, 2024

Reporting Summary

Nature Portfolio wishes to improve the reproducibility of the work that we publish. This form provides structure for consistency and transparency in reporting. For further information on Nature Portfolio policies, see our Editorial Policies and the Editorial Policy Checklist.

Statistics

For all statistical analyses, confirm that the following items are present in the figure legend, table legend, main text, or Methods section.

n/a	Confirmed	
☐	☒	The exact sample size (*n*) for each experimental group/condition, given as a discrete number and unit of measurement
☐	☒	A statement on whether measurements were taken from distinct samples or whether the same sample was measured repeatedly
☐	☒	The statistical test(s) used AND whether they are one- or two-sided<br>*Only common tests should be described solely by name; describe more complex techniques in the Methods section.*
☐	☒	A description of all covariates tested
☐	☒	A description of any assumptions or corrections, such as tests of normality and adjustment for multiple comparisons
☐	☒	A full description of the statistical parameters including central tendency (e.g. means) or other basic estimates (e.g. regression coefficient) AND variation (e.g. standard deviation) or associated estimates of uncertainty (e.g. confidence intervals)
☐	☒	For null hypothesis testing, the test statistic (e.g. *F*, *t*, *r*) with confidence intervals, effect sizes, degrees of freedom and *P* value noted<br>*Give P values as exact values whenever suitable.*
☒	☐	For Bayesian analysis, information on the choice of priors and Markov chain Monte Carlo settings
☒	☐	For hierarchical and complex designs, identification of the appropriate level for tests and full reporting of outcomes
☐	☒	Estimates of effect sizes (e.g. Cohen's *d*, Pearson's *r*), indicating how they were calculated

Our web collection on statistics for biologists contains articles on many of the points above.

Software and code

Policy information about availability of computer code

Data collection	Nikon's NIS-Elements imaging software<br>Typhoon Scanner Control software (Version 5.0)<br>ChemiDoc Touch Imaging System (Bio-Rad)<br>standard Illumina software for the NextSeq 500 and HiSeq 2500 platforms.<br>Libraries were multiplexed and subjected to high-throughput sequencing using a NextSeq 2000 instrument with 50 bp single read runs. Libraries were loaded onto the flow cell at 650 pM final concentration.<br>raw reads were processed to remove eventual sequences of Illumina adapter and, for RiboMethSeq protocol, short reads <40 nt were selected to capture exact positions of both 5'- and 3'-ends. Alignment was performed on the mature sequences of yeast rRNA. After *.bam conversion to *.bed format, both 5'- and 3'-ends (or only 5'-end for HydraPsiSeq) were counted using custom awk script. Combined 5'/3'-end count was used for calculation of RiboMethSeq scores and MethScore (also known as ScoreC2 for +/- 2 nt window) was used for quantification of the methylation level at all known Nm positions in yeast S. cerevisiae rRNA. HydraPsiSeq data were treated in a similar way, but only 5'-end count was used to establish raw U cleavage profile. Normalization to random cleavages observed for A, C and G nucleotides in 10 nt window was used to create NormUcount profile. Non-U nucleotides were dropped and PsiScore (conceptually identical to MethScore used in RiboMethSeq) was used for quantification of the pseudouridylation level.
Data analysis	Fiji: an open-source platform for biological-image analysis. Nature Methods, 9(7), 676–682. doi:10.1038/nmeth.2019.<br>R-studio has been used for genrating box plot<br>MaxQuant software package version 1.6.2.10 for Turbo ID analysis<br>PONDR (Peng et al., 2006)<br>ApE: A plasmid Editor by M. Wayne Davis<br>UniProt: Blast (https://www.uniprot.org/)

Image Lab Software (Bio-rad)
CIDER is a webserver being developed by the Pappu Lab (https://pappulab.wustl.edu/CIDERinfo.html)

For manuscripts utilizing custom algorithms or software that are central to the research but not yet described in published literature, software must be made available to editors and reviewers. We strongly encourage code deposition in a community repository (e.g. GitHub). See the Nature Portfolio guidelines for submitting code & software for further information.

Data

Policy information about availability of data

All manuscripts must include a data availability statement. This statement should provide the following information, where applicable:

- Accession codes, unique identifiers, or web links for publicly available datasets
- A description of any restrictions on data availability
- For clinical datasets or third party data, please ensure that the statement adheres to our policy

Accession codes used in this study are listed in the Data Availability statement, together with their corresponding hyperlink:
PRJEB67499 [https://www.ebi.ac.uk/ena/browser/view/PRJEB67499]
PRJEB67500 [https://www.ebi.ac.uk/ena/browser/view/PRJEB67500]
The MS proteomics data have been deposited to the ProteomeXchange Consortium via the PRIDE partner repository with the dataset identifier PXD056946 [https://www.ebi.ac.uk/pride/archive/projects/PXD056946].

Research involving human participants, their data, or biological material

Policy information about studies with human participants or human data. See also policy information about sex, gender (identity/presentation), and sexual orientation and race, ethnicity and racism.

Reporting on sex and gender	Our study does not include data from human subjects.
Reporting on race, ethnicity, or other socially relevant groupings	Our study does not include data from human subjects.
Population characteristics	Our study does not include data from human subjects.
Recruitment	Our study does not include data from human subjects.
Ethics oversight	Our study does not include data from human subjects.

Note that full information on the approval of the study protocol must also be provided in the manuscript.

Field-specific reporting

Please select the one below that is the best fit for your research. If you are not sure, read the appropriate sections before making your selection.

☒ Life sciences ☐ Behavioural & social sciences ☐ Ecological, evolutionary & environmental sciences

For a reference copy of the document with all sections, see nature.com/documents/nr-reporting-summary-flat.pdf

Life sciences study design

All studies must disclose on these points even when the disclosure is negative.

Sample size	Detailed information for the individual experiments, including exact sample number (n) are stated in the figure legends, the methods section as well as the source data file. Sample size (n) was determined for each set of experiments to reveal reproducibility and/or statistical significance of the data. Sample sizes for RiboMeth-seq eand hydra-psy seq experiments was limited to three or four biological replicates and does not require more due to high reproducibility. Although we assumed a normal distribution of the large (n> 40) microscopy quantification data set, formal tests and validation of this assumption were performed. The method of data normality we used to validate the normal distribution of our samples is the relative value of the standard deviation to the mean. The standard deviation was systematically less than half the mean, indicating that the data are considered normal.
Data exclusions	Raw reads were processed to remove eventual sequences of Illumina adapter and, for RiboMethSeq protocol, short reads <40 nt were selected to capture exact positions of both 5'- and 3'-ends.<br>IDR smaller that 40 amino acids has been excluded in order to assess only the role of large IDRs.<br>No other data were excluded.
Replication	Reproducibility was determined by replications and statistical analyses were appropriate. All attempts at replication were successful.Live imaging was performed from random locations on the coverslips of three biologically independent replicates to minimise the effect of covariates. All comparative experiments were performed using cells showing no major cell growth defect and all experiments were performed with appropriate controls and established conditions to minimise the impact of covariates. Two or more biological replicates were performed

for all experiments (except for mass spectrometry analysis) and all replicates were included in the analyses. The mass spectrometry analysis was performed with appropriate controls and used as an orthogonal approach to confirm our conclusions, initially based on multiple approaches (RNA/ DNA and protein immunoprecipitations, yeast two-hybrids assays and microscopy approaches). Detailed information on individual experiments, including the exact number of samples, are given in the figure legends, the Methods section or in the source data file provided with this paper.

Randomization

Yeast strains used in this study were selected at random from among those generated that contained the correct genotype. Randomization was not performed during biochemical and cell biological experiments.

Blinding

Live imaging was performed from random locations on the coverslips of three biologically independent replicates. For biochemical experiments, blinding is not relevant as analysis biases are very limited for Northern and Western blotting and ChIP but samples were treated in different order for each experiments to avoid any effect due to manipulation hazard. Other blinded experiments were not performed in this study.

Reporting for specific materials, systems and methods

We require information from authors about some types of materials, experimental systems and methods used in many studies. Here, indicate whether each material, system or method listed is relevant to your study. If you are not sure if a list item applies to your research, read the appropriate section before selecting a response.

Materials & experimental systems

n/a	Involved in the study
☐	☒ Antibodies
☐	☒ Eukaryotic cell lines
☒	☐ Palaeontology and archaeology
☒	☐ Animals and other organisms
☒	☐ Clinical data
☒	☐ Dual use research of concern
☒	☐ Plants

Methods

n/a	Involved in the study
☒	☐ ChIP-seq
☒	☐ Flow cytometry
☒	☐ MRI-based neuroimaging

Antibodies

Antibodies used

HA-tagged proteins were detected using HRP-conjugated mouse monoclonal anti-HA antibodies (Roche Diagnostics, Cat. # 12013819001, 1:1000 dilution); GFP-tagged proteins were detected using polyclonal anti-GFP rabbit antibodies (1:1000 dilution) generated by custom antibody production services and kindly provided by Marlène Faubladier and Pierre-Emmanuel Gleizes. RNAPI subunits were detected using rabbit polyclonal antibodies detecting all subunits, kindly provided by Michel Riva. Secondary antibodies were purchased from Promega (HRP-conjugated anti-mouse antibodies, Cat. # W402B, 1:10,000 dilution; HRP-conjugated anti-rabbit antibodies, Cat. # W401B, 1:10,000 dilution). Pgk1 was detected using a monoclonal anti-Pgk1 antibody (Invitrogen, Cat. # 2C5D8, 1:5,000 dilution).

Validation

Commercially available antibodies were validated by the manufacturer. All antibodies are from published studies and were used previously.

HRP-conjugated mouse monoclonal anti-HA antibodies (Roche Diagnostics, Cat. # 12013819001, 1:1000 dilution);
https://www.sigmaaldrich.com/FR/fr/product/roche/12013819001?srsltid=AfmBOooLseduP48wgjF4yjec3DDDYoZr1JdTM7mx0IQtfBQZONFLe1lL

rabbit polyclonal antibodies detecting all subunits, kindly provided by Michel Riva.
https://www.jbc.org/article/S0021-9258(18)43484-9/pdf

anti-GFP rabbit antibodies
https://academic.oup.com/nar/article/40/13/6270/1009704

HRP-conjugated anti-mouse antibodies, Cat. # W402B
https://france.promega.com/products/protein-detection/primary-and-secondary-antibodies/anti_mouse-igg-h-and-l-hrp-conjugate/?catNum=W4021

HRP-conjugated anti-rabbit antibodies, Cat. # W401B
https://france.promega.com/products/protein-detection/primary-and-secondary-antibodies/anti-rabbit-igg-h-and-l-hrp-conjugate/?catNum=W4011

Eukaryotic cell lines

Policy information about cell lines and Sex and Gender in Research

Cell line source(s)	All yeast strains used in this study are based on S. cerevisiae W303.
Authentication	All strains were verified by marker selection, western blotting and/or PCR amplification of specific genomic regions. All plasmids introduced were verified by Sanger sequencing.
Mycoplasma contamination	There is no test for mycoplasma contamination in S. cerevisiae study.
Commonly misidentified lines (See ICLAC register)	Not applicable

Plants

Seed stocks	No plants has been used in this study
Novel plant genotypes	No plants has been used in this study
Authentication	No plants has been used in this study
